# Histopathological techniques for the diagnosis of combat-related invasive fungal wound infections

**DOI:** 10.1186/s12907-016-0033-9

**Published:** 2016-07-07

**Authors:** Sarah M. Heaton, Amy C. Weintrob, Kevin Downing, Bryan Keenan, Deepak Aggarwal, Faraz Shaikh, David R. Tribble, Justin Wells, the Infectious Disease Clinical Research Program Trauma Infectious Disease Outcomes Study Group

**Affiliations:** Department of Pathology, Walter Reed National Military Medical Center, 8901 Wisconsin Avenue, Bethesda, MD 20814 USA; Infectious Disease Clinical Research Program, Department of Preventive Medicine and Biostatistics, Uniformed Services University of the Health Sciences, Bethesda, MD USA; The Henry M. Jackson Foundation for the Advancement of Military Medicine, Inc., Bethesda, MD USA

**Keywords:** Invasive fungal infections, Invasive mold infections, Combat-related infections, Histopathology, Histochemical stains for fungus

## Abstract

**Background:**

Effective management of trauma-related invasive fungal wound infections (IFIs) depends on early diagnosis and timely initiation of treatment. We evaluated the utility of routine staining, histochemical stains and frozen section for fungal element identification.

**Methods:**

A total of 383 histopathological specimens collected from 66 combat-injured United States military personnel with IFIs were independently reviewed by two pathologists. Both periodic acid-Schiff (PAS) and Gomori methenamine silver (GMS) stains were used on 74 specimens. The performance of the two special stains was compared against the finding of fungal elements via any histopathological method (ie, special stains or hematoxylin and eosin). In addition, the findings from frozen sections were compared against permanent sections.

**Results:**

The GMS and PAS results were 84 % concordant (95 % confidence interval: 70 to 97 %). The false negative rate of fungal detection was 15 % for GMS and 44 % for PAS, suggesting that GMS was more sensitive; however, neither stain was statistically significantly superior for identifying fungal elements (*p* = 0.38). Moreover, 147 specimens had frozen sections performed, of which there was 87 % correlation with permanent sections (60 % sensitivity and 98 % specificity). In 27 permanent sections, corresponding cultures were available for comparison and 85 % concordance in general species identification was reported.

**Conclusions:**

The use of both stains does not have an added benefit for identifying fungal elements. Furthermore, while the high specificity of frozen section may aid in timely IFI diagnoses, it should not be used as a stand-alone method to guide therapy due to its low sensitivity.

## Background

Trauma-related invasive fungal wound infections (IFIs) are characterized by high mortality (up to 38 %) and substantial morbidity (eg, surgical amputations). Consequently, early diagnosis and timely initiation of treatment are critical for the successful management of the disease [1–7]. While use of cultures to identify specific pathogens remains an effective diagnostic method, fungal organisms may take weeks to grow, if they grow at all. Therefore, IFIs are often diagnosed through routine histopathological examination of tissue specimens, which is a process that can be completed and interpreted within 24-48 h when expedited [8–11]. Although histopathology is useful for differentiating between fungal colonization and infection through the identification of tissue invasion or inflammation [8], ascertainment of the organism’s genus and species is limited [9, 12, 13].

The most common type of histopathological examination involves using permanent (paraffin-embedded) sections of biopsy tissue stained with hematoxylin and eosin (H&E). Although H&E is capable of staining the fungal cell wall, it is easy to overlook fungal organisms due to limited staining differential from background tissue. Moreover, when fungal organisms are observed with H&E, it can be difficult to identify characteristics useful for classification (eg, septate versus aseptate) [9, 14]. Therefore, if an IFI is suspected, but organisms are either not identified or are poorly visualized with H&E, the use of additional stains, particularly Gomori methenamine silver (GMS) and/or periodic acid-Schiff (PAS), can be used to either rule out a fungal infection or identify morphologic characteristics. Both GMS and PAS stains provide greater contrast by highlighting the fungal cell wall; however, misidentification, false-positives and false-negatives, do occur with these techniques. Specifically, GMS may result in poor staining when there is fragmentation or necrosis of fungal elements [9]. A further limitation is that GMS masks the natural color of mold, making it difficult to identify hyaline organisms from dematiaceous [15]. When PAS is used, background tissue components may also be stained along with the fungal cell wall [9, 16].

Another form of histopathological examination is an intraoperative consultation or frozen section, which provides a more rapid result than permanent sections. This technique involves freezing a portion or the entire debridement specimen, which allows for expedient cutting, slide mounting, and staining. Typically, these results can be obtained within twenty minutes of tissue submission from the operating room. Nonetheless, this expedited turn-around time comes at a cost as the histological quality is significantly compromised [17].

During the recent war in Afghanistan, clinicians observed a higher than expected proportion of combat-related IFIs among wounded United States (U.S.) military personnel [7]. In general, diagnosis of the combat-related IFIs was not dependent upon mold growth from wound cultures, but more commonly the result of identification of fungal elements on histologic examination. Overall, 70 % of IFI patients were diagnosed in this manner [18].

There is limited literature regarding the use of frozen and permanent sections for the diagnosis of IFIs in this setting. The use of GMS and PAS as supplemental staining also needs further elucidation. Our objective was to survey pathologic information collected from U.S. military personnel with combat-related IFIs to determine the utility of GMS and PAS stains for IFI diagnosis. We also examined the role of frozen sections in aiding the diagnosis of IFIs. Lastly, we evaluated the correlation between fungal morphology, as observed with light microscopy, and culture-based identification.

## Methods

### Study population

Data were collected from U.S. military personnel with combat-related injuries sustained between June 1, 2009 and August 31, 2011 and medically evacuated to Landstuhl Regional Medical Center (LRMC) in Germany. Following initial treatment, patients were subsequently transferred to a participating military hospital in the U.S.: Walter Reed Army Medical Center in Washington, DC, National Naval Medical Center in Bethesda, MD, and Brooke Army Medical Center in San Antonio, TX. The overarching project is the U.S. Department of Defense (DoD) – Department of Veterans Affairs, Trauma Infectious Disease Outcomes Study (TIDOS), which collects longitudinal, prospective data in order to analyze infectious complications among service members with deployment-related trauma [19]. The study was approved by the Infectious Disease Institutional Review Board of the Uniformed Services University of the Health Sciences.

### Invasive fungal infection case identification

The IFI patients were identified from the population of military personnel with combat-related trauma based upon evidence of recurrent wound necrosis after a minimum of two successive surgical debridements and fungal infection of viable tissue on either histopathology or culture [7, 18]. Histopathologic evidence included either fungal angioinvasion and/or fungal elements seen on routine histochemical staining (H&E and/or special stains). Inclusion in the study population also required a review of surgical pathology slides at the time of diagnosis.

Demographics and injury characteristics of patients who met the IFI case criteria were collected from the DoD Trauma Registry [20], while fungal culture and histopathological data were obtained through the supplemental TIDOS infectious disease module. Histopathology specimens associated with IFI patients were independently reviewed by two surgical pathologists. Furthermore, infectious disease and trauma surgery services case records were also examined.

### Invasive fungal wound infection pathology

Tissue samples collected from U.S. military personnel with combat-related trauma IFIs were retrospectively examined to compare sensitivity and specificity of staining and preparatory methods. For each specimen with frozen sections, results were obtained from the final pathology report. All specimens required slides prepared with H&E as per routine surgical pathology handling. The GMS and/or PAS stains were performed on the specimen per primary pathologist discretion at time of diagnosis. Artisan GMS and PAS-green stain kits (Dako, Carpinteria, CA) were used with Dako Artisan Link Special Staining System. After case completion, all slides were blindly and individually re-read by two pathologists, who upon disagreement on any data element would reach consensus during a multi-headed microscope review. Presence of invasive fungus, identification characteristics (described below), tissue type involved, presence of necrosis and special staining result were recorded for each case. A threshold for special stain positivity was set as easily discernible fungal wall staining as compared to background at 100x magnification. This threshold simplified interpretation and provided more clinically relevant staining data. Limited fungal identification was utilized as previously defined, including categories of aseptate, septate, yeast, or polymicrobial [9]. When available, histopathology was compared against results from cultures collected from the same wound site.

### Statistical analysis

Due to the primary mechanism of injury (ie, dismounted blast), polytrauma was common among combat casualties and; therefore, multiple wounds with IFI may develop in an individual patient. Independency was assumed among all specimens, including those collected from the same patient (ie, specimens that were not collected from the same wound site on the same day). Stain performance was demonstrated by sensitivity for fungal detection of the GMS and PAS staining methods. The presence of fungal elements identified via any histopathological method (ie, GMS, PAS, and/or H&E) was used as the reference for the evaluation. Similarly, frozen identification was compared to permanent sections. Lastly, descriptions of fungal morphology reported by pathologists following examination of histologic specimens were compared to results of concurrent fungal cultures (±3 days) taken from the same anatomic site. All comparisons were conducted using McNemar’s test and Kappa’s coefficient. Statistical analysis was conducted using SAS® version 9.3 (SAS, Cary, NC) and significance was defined as *p* < 0.05.

## Results

### Patient demographics and injury characteristics

**Fig. 1:**
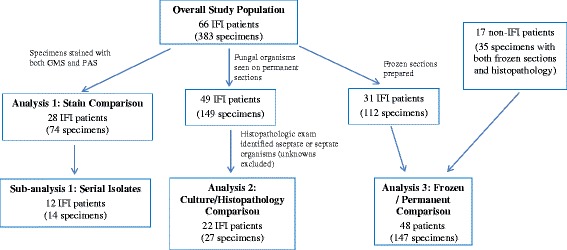
Flow diagram of study population through the three separate analyses. Patients were included in the analysis if they had a review of surgical pathology slides at the time of diagnosis

From the population of wounded U.S. military personnel admitted to LRMC (June 2009 - August 2011), 66 IFI patients met the criteria for inclusion in the study population (ie, availability of surgical pathology slides reviewed at the time of diagnosis). The characteristics of these IFI cases have been previously described [7, 18]. All of the patients were young men (median age of 23 years) and the majority sustained moderate to severe injuries as indicated by the injury severity score (83 % ≥16), which is a summary score estimated from injury values determined for the six main body regions [21]. Tissue specimens were examined in separate study population subset analyses based on the following criteria (Fig. 1): stained with both PAS and GMS (Analysis 1); having aseptate or septate organisms identified on permanent sections (Analysis 2); and having frozen sections available (Analysis 3).

### Invasive fungal infection surgical pathology findings

A total of 383 specimens were collected from the 66 IFI patients (141 wound sites), of which 149 specimens (49 patients) displayed fungal organisms on permanent sections (ie, paraffin-embedded tissues stained for examination). Specimens sent for pathological examination were largely comprised of more than one type of tissue with the predominance containing fibroconnective tissue (91 %) followed by adipose (75 %), muscle (55 %), skin (17 %), and bone (7 %). Moreover, specimen sites were largely extremity wounds with 54 % of tissue samples collected from the lower extremities and 15 % from the upper. The remaining specimens were collected from the pelvis/hip (27 %), abdomen (4 %), and craniofacial area (0.3 %).

### Staining methods (analysis 1)

**Fig. 2:**
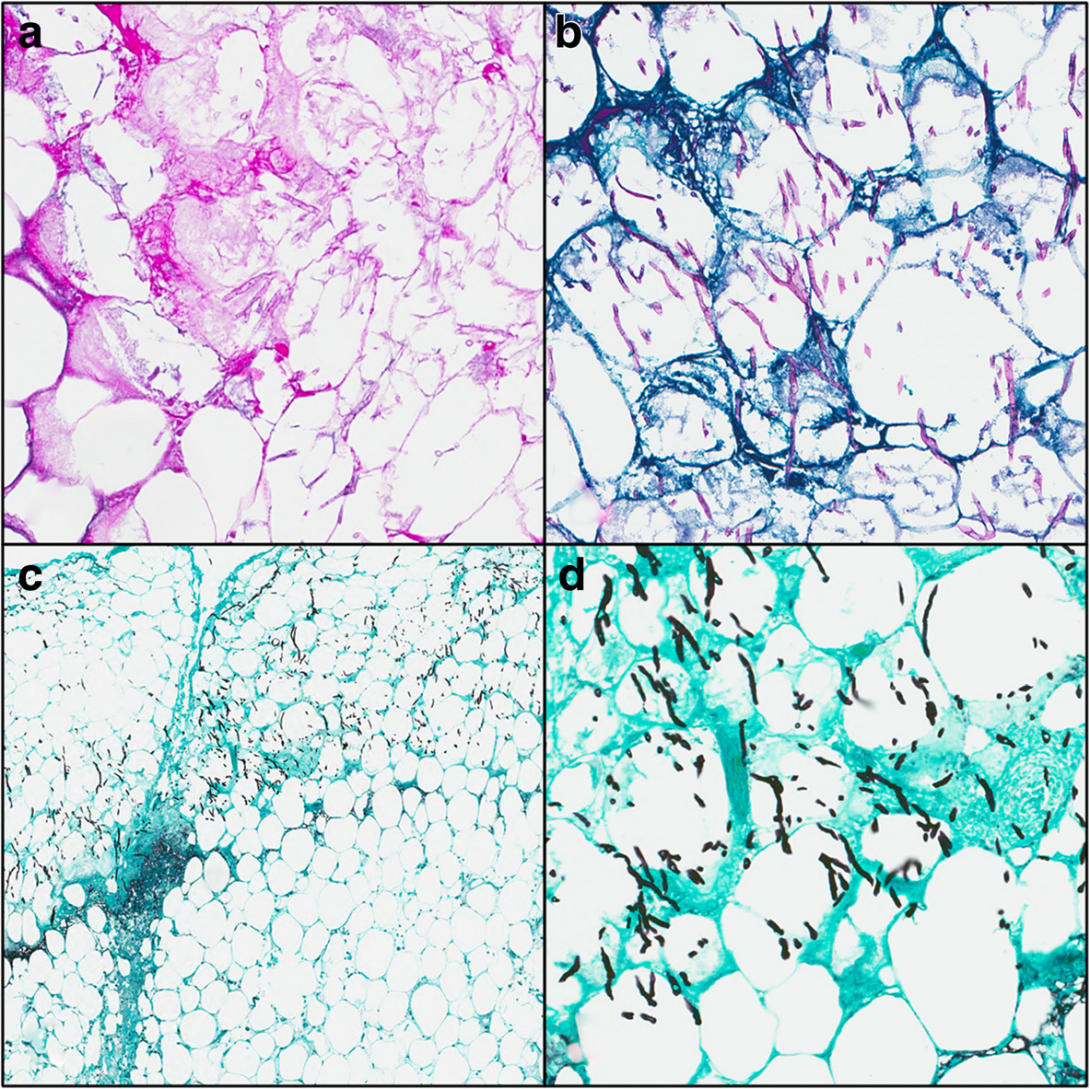
Necrotic fibroadipose tissue with fungal organisms consistent with septate, acute angle branching morphology. (**a**) hematoxylin and eosin stain, 20X; (**b**) Periodic Acid-Schiff stain, 20X; (**c**) Gomori Methenamine Silver stain, 5X; (**d**) Gomori Methenamine Silver stain, 20X

**Fig. 3:**
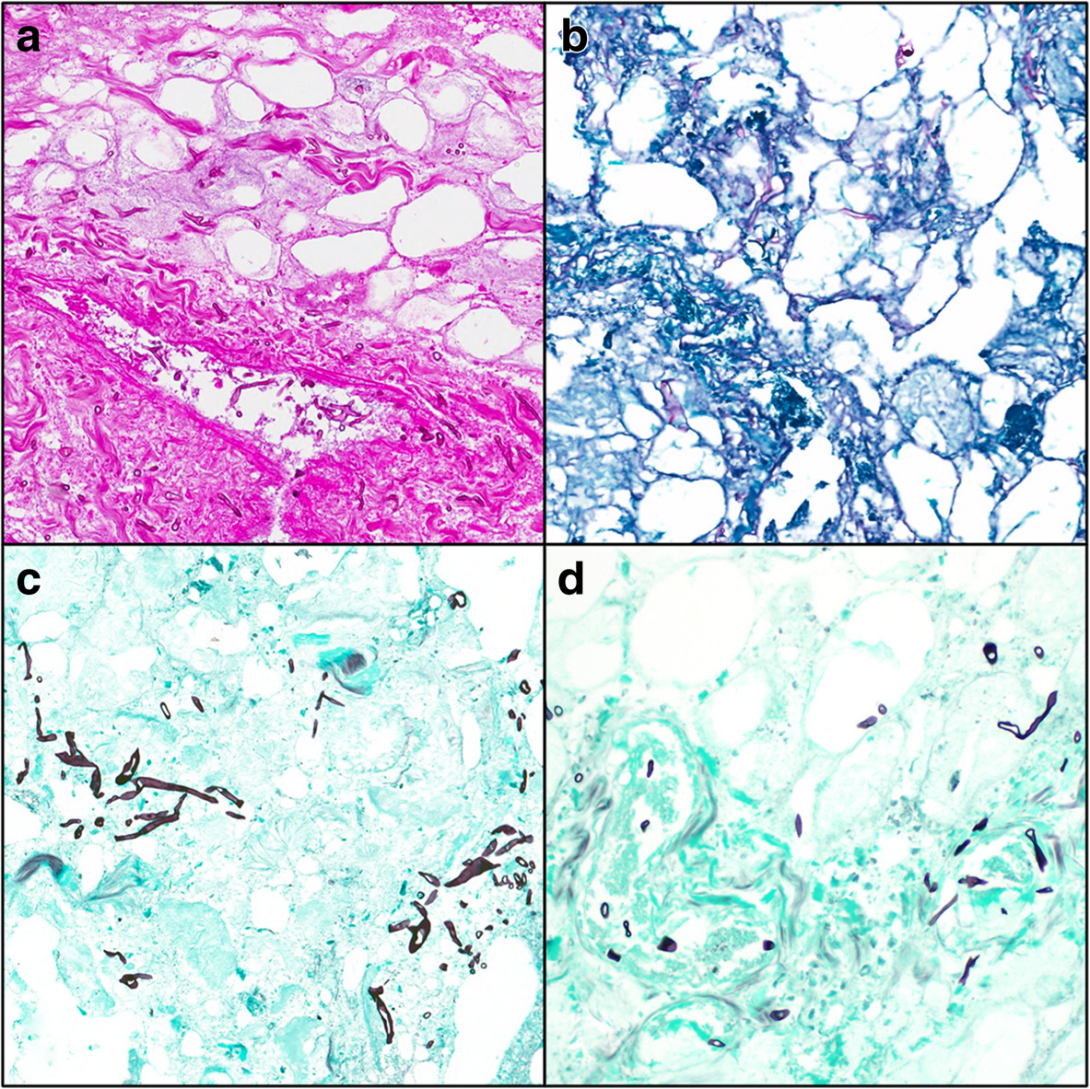
Necrotic fibroadipose tissue with fungal organisms consistent with aseptate Zygomycete species (broad, ribbon-like hyphae). Angioinvasion can be seen in parts A and D. (**a**) hematoxylin and eosin stain, 20X; (**b**) Periodic Acid-Schiff stain, 20X; (**c** and **d**) Gomori Methenamine Silver stain, 20X

All 383 tissue specimens were stained with routine H&E along with at least one of the special stains. An example of tissue with different stains with resulting visualized fungal organisms consistent with mold classified as septate and aseptate are presented in Figs. 2 and 3. For this subset analysis, 74 specimens (28 patients) that were stained with both PAS and GMS were examined (Table 1). Both stains decorated fungal elements in 20 (27 %) specimens, while fungal elements were not present with either stain (or H&E) in 49 (66 %) specimens. Of the five discordant results, one (1 %) specimen had fungal elements highlighted only with PAS stain and another four (5 %) only with GMS. Overall, there were no significant differences between the staining methods for visualizing fungal elements (*p* = 0.38) with concordance estimated to be approximately 84 % with a 95 % confidence interval range between 70 to 97 %. When GMS used alone was compared to GMS used in combination with PAS (Table 2), there was no significant difference in fungal detection (*p* = ~1.0).

**Table 1:** Comparison of results with PAS and GMS staining for the identification of fungal elements^a^

Results with PAS stain	Results with GMS stain		
	Positive for fungal elements	Negative for fungal elements	Total
Positive for fungal elements	20	1	21
Negative for fungal elements	4	49	53
Total	24	50	74

^a^ Kappa coefficient for comparison is 84 % (95 % confidence interval of 70-97 %); McNemar’s test p-value =0.38

**Table 2:** Comparison of GMS staining alone versus PAS with GMS staining for identification of fungal elements^a^

	GMS Plus PAS staining		
GMS alone	Positive for fungal elements	Negative for fungal elements	Total
Positive for fungal elements	24	0	24
Negative for fungal elements	1	49	50
Total	25	49	74

^a^ Kappa coefficient for comparison is 97 % (95 % confidence interval of 91-100 %); McNemar’s test p-value ~1.0

Among 12 patients (14 wound sites), serial isolates were collected from the same wound site at multiple time points. An evaluation of these serial specimens (total of 14) found that two initially negative wound sites had fungal elements in subsequent biopsies with GMS; however, fungal elements were not visualized at any time using PAS stain for these specimens. In addition, one wound site that was initially negative with GMS had fungal elements in later tissue samples, while PAS stain identified fungal elements in both the initial and subsequent specimens. Using the total number of cases with fungal elements identified on histopathology (ie, H&E plus GMS and/or PAS) as the point of reference, the sensitivity for fungal detection was 56 % and 85 % for PAS and GMS, respectively, which translates to a false negative rate of 44 % and 15 %, respectively.

### Mycology and histopathologic morphology comparison (analysis 2)

The histopathological findings of 27 specimens identified the mold as either aseptate, septate, or mixed (both septate and aseptate), which allowed for comparison of fungal morphology against tissue cultures collected from the same wound site (more than 55 % of wound cultures did not grow mold). Specimens with histopathological findings that could not clearly designate the mold as aseptate, septate, or mixed or did not have a corresponding tissue culture that grew mold were excluded from this analysis.

Among the wound cultures that grew mold, the following were identified: *Acrophialophora fusispora*, *Aspergillus* spp., *Fusarium* spp., *Graphium* spp., *Mucor* spp., and *Mycelia sterilia*. Between the permanent sections and cultures, there was correlation in 85 % (23 of 27) of the wounds (90 % in histopathologically classified septate organisms and 72 % in aseptate) (Table 3). Six histopathological specimens and three microbiological cultures had ‘mixed’ findings. Although the corresponding culture and histopathology specimens always observed either septate or aseptate only, they were counted as concordant because at least one of the organisms from the mixed findings was identified.

**Table 3:** Comparison of results with permanent sections and fungal culture for the identification of fungal morphology

Morphology with permanent sections	Morphology with cultures			
	Non-*Mucorales* and septate fungi	*Mucorales*	Mixed (both septate and aseptate)	Total
Septate assumed non-*Mucorales*	9	1	0	10
Aseptate assumed *Mucorales*	3	5	3^a^	11
Mixed (both septate and aseptate)	3^a^	3^a^	0	6
Total	15	9	3	27

^a^ ‘Mixed’ findings were counted as concordant with the ‘septate/asepate only’ results because at least one of the organisms observed in the specimens designated as mixed was identified in the corresponding histopathology specimens/cultures

### Frozen sectioning (analysis 3)

**Fig. 4:**
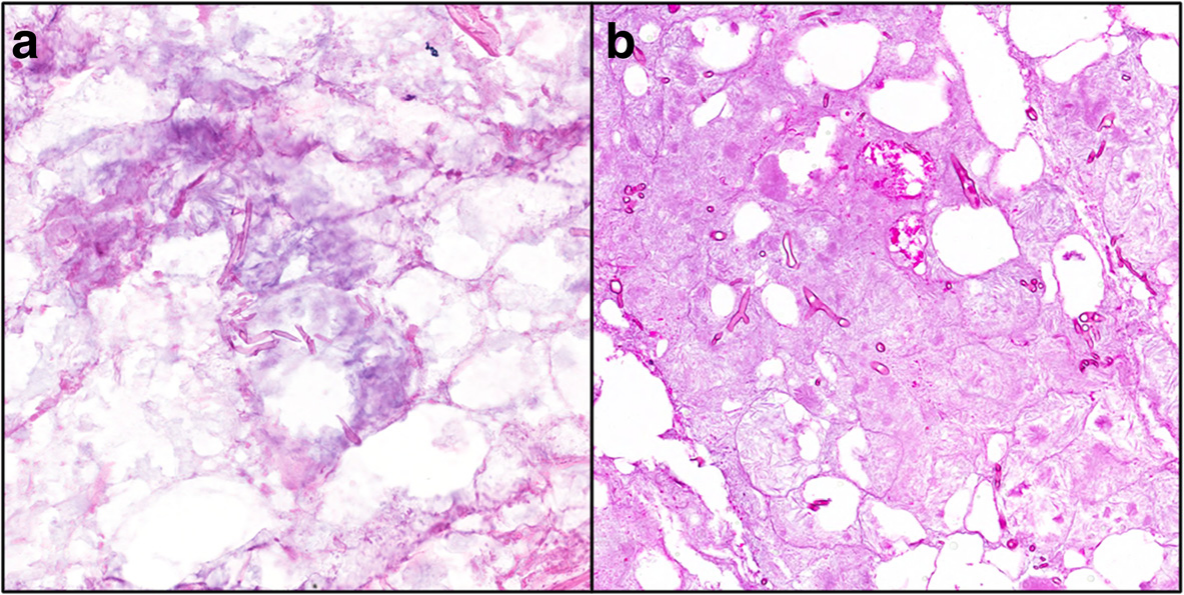
Necrotic fibroadipose tissue with fungal organisms consistent with Zygomycete species (broad, ribbon-like hyphae). (**a**) Frozen section with hematoxylin and eosin stain, 20X; and (**b**) Corresponding permanent section with hematoxylin and eosin stain, 20X

Frozen sections were prepared on 147 specimens collected from 48 patients (Table 4). Fungal elements were identified in 24 (16 %) specimens on both frozen and permanent sections, while fungal elements were not visualized with either method in 105 (71 %) specimens. Using results of the permanent sections as the point of reference (40 with fungal elements and 107 with no visualized fungal elements), the positive and negative predictive values of frozen sections were 92 % and 87 %, respectively (Fig. 4). The sensitivity of frozen section was calculated to be 60 % and specificity was 98 %.

**Table 4:** Comparison of results with frozen and permanent sections for the identification of fungal elements^a^

Results with frozen sections	Results with permanent sections		
	Positive for fungal elements	Negative for fungal elements	Total
Positive for fungal elements	24	2	26
Negative for fungal elements	16	105	121
Total	40	107	147

^a^ The specimens are collected from 48 patients, of which 31 were diagnosed with IFIs and 17 were non-IFI patients

Of the 40 permanent sections with fungal elements, 20 specimens were identified to have aseptate growth, 11 with septate, and 9 with unspecified or multiple molds. Frozen sections identified 13 specimens with aseptate growth (35 % false negative rate), 4 with septate growth (64 % false negative rate), and 7 with unspecified/multiple molds.

## Discussion

Between 2009 and 2011, 6.8 % of combat casualties admitted to LRMC and transferred to a TIDOS-participating hospital were diagnosed with IFIs [22]. A concentrated effort was made to promote earlier diagnosis, which involved collection of tissue samples for culture and histopathological examination from all patients considered to be at risk due to their mechanism and pattern of injury [23]. Although it is agreed that early diagnosis of IFIs is a crucial step in achieving a beneficial patient outcome, there is little experience with routine histopathology techniques in this setting. Therefore, our goal was to compare the utility of two special stains used for visualizing fungal elements, along with examining the results of frozen sections and cultures when compared with permanent section.

While our overall concordance is 85 %, disagreement between the results of morphology on slides and cultures is not uncommon. In general, accuracy of microscopic identification of fungal species using either histopathological or cytological specimens has been estimated to range from 20 % to 80 % [8, 9]. Morphology consistent with *Aspergillus* spp. is hyaline, thin (3-12 μm) septate hyphae with acute angle branching, while the order *Mucorales* is characterized by hyaline, wide (5-20 μm), thin-walled, aseptate, ribbon-like hyphae with right angle branching [9]. Nonetheless, *Aspergillus* spp. may occasionally display morphology consistent with the *Mucorales* order and vice versa resulting in misclassification [8, 9]. In particular, misclassification involving septate and aseptate hyphae may occur in situations with substantial necrosis as the hyphae may become swollen or distorted, making histopathological identification difficult. Moreover, fungal hyphae may be scant, folded, kinked, or fragmented so an accurate description of septation or branching is difficult [8]. Culture also has its limitations as results may be hindered by contamination and the inability to determine colonization from true infection without associated clinical indicators [13, 24]. The duration (ranging from days to weeks) required for mold growth and identification also severely limits use of cultures in acute situations where delay in treatment can result in poor outcomes. These results and the obvious limitations identified need to be considered by our clinical colleagues as they make treatment decisions.

We also evaluated the potential benefit of frozen section preparations in relation to IFI diagnostics [8, 9, 25]. Our data suggest that there is a finite role in identification of fungal organisms at the time of surgery. Specifically, when organisms are identified on frozen sections, this strongly supports an IFI diagnosis. In contrast, the usefulness of a negative result is questionable and should not be used to rule out an IFI if there is a strong clinical suspicion. Large false negative rates are not unexpected as this procedure suffers from inferior histology and is susceptible to sampling error [8, 25]. Unlike permanent sections where the entire tissue sample can be examined, frozen sections often only evaluate selected areas of a larger debridement specimen. Characterization as septate versus aseptate is also problematic as distorted histology and frozen artifacts makes this challenging diagnosis even more difficult. For example, freezing tends to balloon tissues, which may cause a thin hyphae to become broad, leading to inaccuracies [17]. Overall, the findings observed in this military setting are comparable with other publications, which found frozen sections to be largely predictive of IFIs in patients with rhinosinusitis [15, 25, 26]. Furthermore, an assessment of 33 specimens collected from a patient with fungal-infected bedsores found 68 % sensitivity and 100 % specificity of frozen sections in the soft-tissue margin evaluation in wound debridement [27], which is comparable to our findings.

During the combat-related IFI outbreak, pathologists involved with diagnosis often recommended that treatment decisions not be determined by special stains alone. Our findings support this belief. The ease of identification on GMS and PAS may cause pathologists to overlook or quickly review H&E slides. In actuality, rare cases early in the outbreak of IFIs were missed when special stains failed to highlight organisms. When the corresponding H&E was reviewed, organisms were readily found. In our analysis, PAS and GMS had false negative rates of 44 % and 15 %, respectively.

Methods of histologic evaluation to support diagnosis of the disease rapidly evolved following recognition of the unexpected outbreak of IFIs among wounded military personnel. Staining methods initially included both GMS and PAS to increase sensitivity; however, due to anecdotal evidence, pathologists began to reduce their reliance on PAS as they believed it was not as sensitive as GMS and added little additional benefit. Furthermore, the decision to focus on one stain was also based on factors related to cost and labor required to perform multiple stains on each tissue block. Our results demonstrated a high level of concordance between the special stains with GMS more sensitive in this series (GMS false negative rate of 15 % versus 44 % with PAS), indicating that GMS may be more suitable for identifying zygomycetes. Supporting this initial decision, the data also demonstrate that there is no significant benefit to using both stains in an effort to identify fungal elements. Therefore, we recommend use of GMS for fungal identification over PAS.

Routine histopathologic techniques and culture, as reported in our study, are still the most commonly used techniques for rapid fungal identification. Nonetheless, in our opinion, we believe that the future of early diagnosis with accurate speciation may ultimately be achieved through more complex testing in conjunction with histopathology, to include immunohistochemistry, in situ hybridization, PCR and Matrix-assisted laser desorption/ionization. These tests should be able to quickly and definitively identify fungal species in tissues and may better detect dual infections [9]. Investigations to determine the benefit of these modalities are underway.

While our clinical setting, specific to blast injuries sustained by soldiers serving in Afghanistan, seems narrow, we believe these data are applicable to other presentations. Recent examples of invasive fungal infections in the civilian setting include the occurrence of trauma-related IFIs following the Joplin tornado and the outbreak of mucormycosis associated with linens in a pediatric hospital [2, 28]. We hope that our report provides insight for pathologists and clinicians into a truly unfortunate problem.

## Conclusions

Overall, the GMS and PAS staining for the identification of invasive fungal elements were 84 % concordant and neither stain was statistically significantly better with identifying fungal elements (*p* = 0.38). Despite their obvious advantages for supporting diagnosis of fungal infections, GMS and PAS had false negative rates for fungal detection of 15 % and 44 %, respectively, which stresses the importance of closely reviewing the H&E. Furthermore, the authors demonstrated that while frozen section had high specificity (98 %), it should not be used as a standalone method for the diagnosis of invasive fungal wound infections due to its low sensitivity.

## Abbreviations

DoD, Department of Defense; GMS, Gomori methenamine silver; H&E, Hematoxylin and eosin; IFI, Invasive fungal wound infections; LRMC, Landstuhl Regional Medical Center; PAS, periodic acid-Schiff; TIDOS, Trauma Infectious Disease Outcomes Study; U.S., United States
